# SAHA and/or MG132 reverse the aggressive phenotypes of glioma cells: An *in vitro* and vivo study

**DOI:** 10.18632/oncotarget.13680

**Published:** 2016-11-29

**Authors:** Xue-feng Yang, Zhi-juan Zhao, Jia-jie Liu, Xiang-hong Yang, Yang Gao, Shuang Zhao, Shuai Shi, Ke-qiang Huang, Hua-chuan Zheng

**Affiliations:** ^1^ Cancer Center, The First Affiliated Hospital of Jinzhou Medical University, Jinzhou 121001, China; ^2^ Department of Pathology, Shengjing Hospital of China Medical University, Shenyang 110004, China; ^3^ Department of Stomatology, The Second Affiliated Hospital of Jinzhou Medical University, Jinzhou 121001, China; ^4^ Life Science Institute of Jinzhou Medical University, Jinzhou 121001, China

**Keywords:** glioma, suberoylanilide hydroxamic acid, histone acetylation, MG132, chemotherapy

## Abstract

To elucidate the anti-tumor effects and molecular mechanisms of SAHA (a histone deacetylase inhibitor) and MG132 (a proteasome inhibitor) on the aggressive phenotypes of glioma cells, we treated U87 and U251 cells with SAHA or/and MG132, and detected phenotypes’ assays with phenotype-related molecules examined. It was found that SAHA or/and MG132 treatment suppressed proliferation in both concentration- and time-dependent manners, inhibited energy metabolism, migration, invasion and lamellipodia formation, and induced G_2_ arrest and apoptosis in the glioma cells. The treatment with SAHA increased the expression of acetyl-histones 3 and 4, which were recruited to the promoters of *p21, p27, Cyclin D1, c-myc* and *Nanog* to down-regulate their transcriptional levels. Expression of acetyl-histones 3 and 4 was higher in gliomas than normal brain tissues. Both drugs’ exposure suppressed tumor growth in nude mice by inducing apoptosis and inhibiting proliferation, but increased serum aminotransferase and creatinine. These results indicated that SAHA and/or MG132 may suppress the aggressive phenotypes of glioma cells. They might be employed to treat the glioma if both hepatic and renal injuries are prevented.

## INTRODUCTION

Gliomas are the most frequently occurring primary brain tumors in adults [1]. Its incidence has been increasing in the elderly population [2]. In spite of recent important advances in basic molecular mechanisms of gliomas, effective improvements in imaging, surgery, and radiotherapy, as well as the discovery of new promising drugs and targeted agents, its overall prognosis remains poor.

Histone acetyl transferases (HATs) and histone deacetylases (HDACs) are targets for cancer therapy. They determine the acetylation status of histones, the chromatin structure and gene expression. SAHA is the first histone deacetylase inhibitor (HDACi) drug, approved by Food and Drug Administration (FDA) of USA. It has multiple cellular effects and inhibits class I, II and IV HDACs, including the cytoplasmic HDAC6, a member of class IIb [3, 4]. SAHA has the permeability to cross the blood brain barrier and cause biological responses in the mouse brain, therefore making it as a preferred candidate drug for testing in gliomas [5].

A major pathway of intracellular protein degradation includes the proteasome, a multi-subunit enzyme complex that resides in the cytosol and nucleus [6]. MG132 is a proteasome inhibitor that induces apoptosis in tumor cells. The combination of proteasome inhibitors with some anti-tumor drugs such as SAHA comprises a new emerging field in oncology. Therefore, we observed the anti-tumor effects and relevant molecular mechanisms of SAHA or/and MG132 on the aggressive phenotypes of glioma cells *in vitro* and vivo. To clarify the clinicopathological significance of acetyl-histones 3 and 4, we determined their expression in normal brain tissues and glioma samples by immunohistochemistry, and compared them with clinicopathological parameters of gliomas.

## RESULTS

### The effects of SAHA or/and MG132 on the aggressive phenotypes of glioma cells

**Figure 1:**
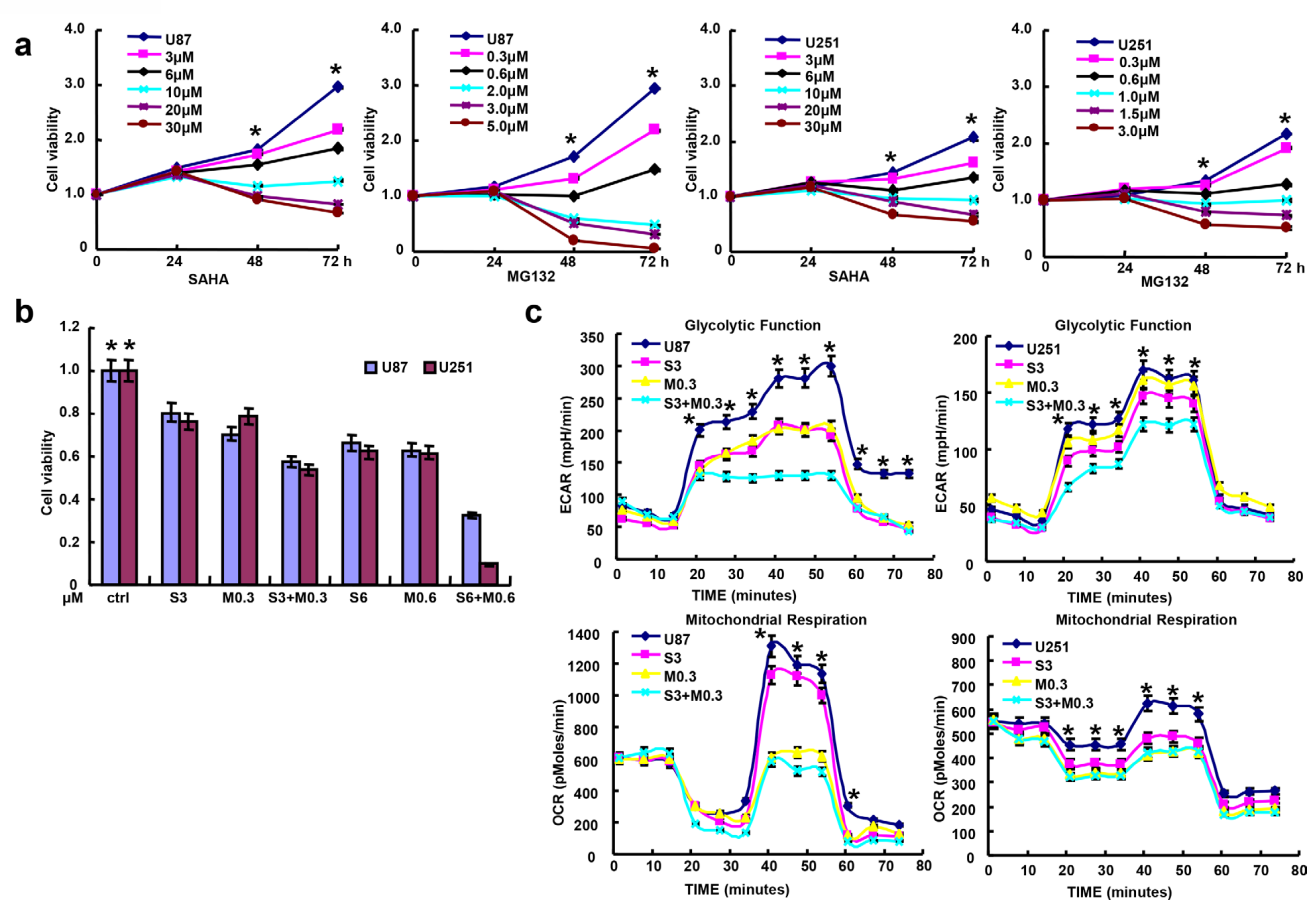
Effects of SAHA or/and MG132 on the proliferation and cellular energy metabolism of glioma cells MTT assays showed that SAHA or MG132 treatment suppressed the proliferation of U87 and U251 cells in either a concentration- or time-dependent manner **a**. with an additive effect **b**. Cellular energy metabolism assay was performed after cells were treated with both drugs for 48 h **c**. Results are representative of 3 different experiments, and the data is expressed as mean ±standard deviation with the control as “1”. Note: S3, SAHA 3 μM; M0.3, MG132 0.3 μM; S6, SAHA 6 μM; M0.6, MG132 0.6 μM; S3 + M0.3, SAHA 3 μM and MG132 0.3 μM; S6 + M0.6, SAHA 6 μM and MG132 0.6 μM. *p< 0.05, vs treatment groups.

**Figure 2:**
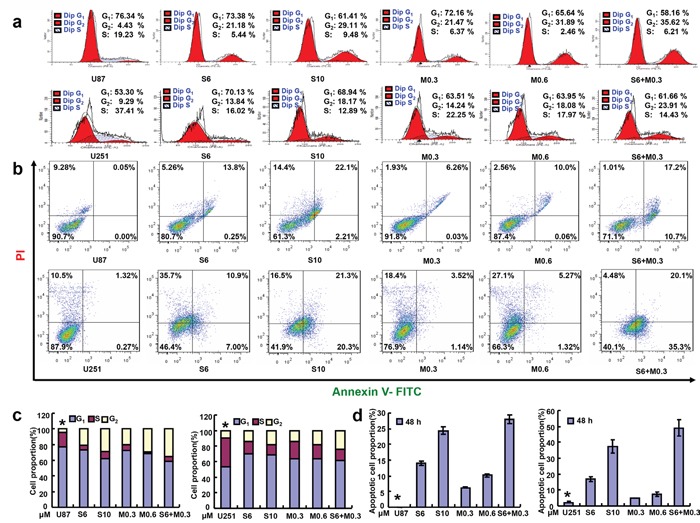
Effects of SAHA or/and MG132 on the cell cycle and apoptosis of glioma cells Flow cytometric analyses of glioma cell lines after PI staining showed that SAHA or/and MG132 treatment induced G_2_ arrest in a concentration-dependent manner in U87 and U251 cells after 48 h **a, c**. SAHA or/and MG132 exposure results in higher levels of apoptosis in U87and U251cells after 48 h **b, d**. Results are representative of 3 different experiments, and the data is expressed as mean ±standard deviation. Note: S6, SAHA 6 μM; M0.3, MG132 0.3 μM; S10, SAHA 10 μM; M0.6, MG132 0.6 μM; S6 + M0.3, SAHA 6 μM and MG132 0.3 μM. *p < 0.05, vs treatment groups.

**Figure 3:**
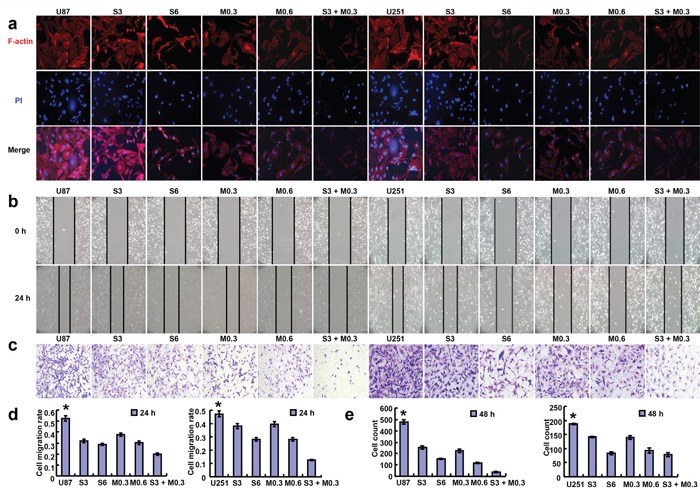
Effects of SAHA or/and MG132 on the migration and invasion of glioma cells The lamellipodia formation in glioma cells was evaluated by immunofluorescence as indicated by F-actin structure after 48 h **(a, 400×)**. Wound healing assays showed that SAHA or/and MG132 treatment decreased the ability of U87 and U251 cells to migrate in a concentration-dependent manner **b, d**. and reduced the invasive potential of U87 and U251 cells **(c, e, 200×)**. Results are representative of 3 different experiments, and the data is expressed as mean ±standard deviation. Note: S3, SAHA 3 μM; M0.3, MG132 0.3 μM; S6, SAHA 6 μM; M0.6, MG132 0.6 μM; S3 + M0.3, SAHA 3 μM and MG132 0.3 μM. * p < 0.05, vs treatment groups.

According to MTT assay, SAHA and MG132 reduced proliferation of the glioma cell lines in either time- or dose-dependent manner (Figure 1a, p<0.05). Additionally, there was an additive effect of SAHA and MG132 on the proliferative inhibition of glioma cells (Figure 1b, p<0.05). SAHA and MG132 can effectively inhibit the energy metabolism of both cells lines (Figure 1c, p<0.05). SAHA or/and MG132 treatment induced G_2_ arrest and apoptosis in both U87 and U251 cells in a concentration-dependent manner (Figure 2a-2d, p<0.05). SAHA or/and MG132 exposure suppressed lamellipodia formation in both glioma cells, as indicated by the loss of F-actin structure (Figure 3a). The wound healing and matrigel transwell invasion assays showed that SAHA or/and MG132 decreased cell migration and invasion (Figure 3b-3e, p<0.05). In addition, SAHA and MG132 acted an additive effect to cause cycle arrest, induce apoptosis, and inhibit cell migration, invasion, lamellipodia formation and cellular energy metabolism in U87 and U251 cells.

### The mRNA and protein expression of phenotype-related molecules in glioma cells after the exposure to SAHA or/and MG132

**Figure 4:**
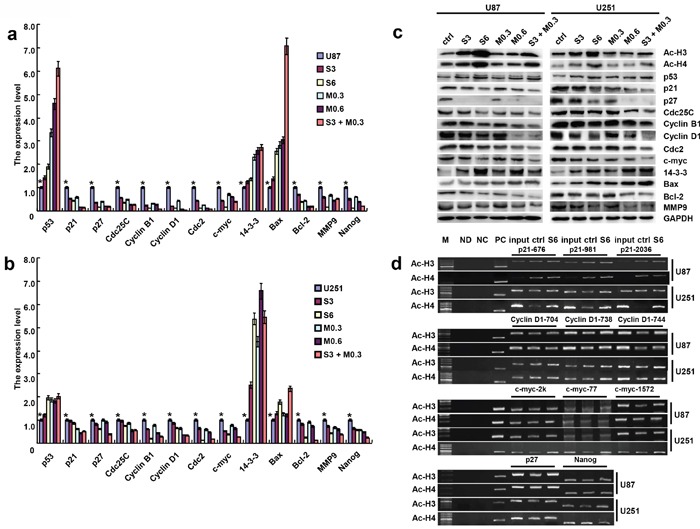
The mRNA and protein expression of gliomas cells treated with SAHA or/and MG132 After the treatment with SAHA or/and MG132, the expression levels of phenotype- related molecules were screened in U87 and U251 cells by real-time RT-PCR **a, b**. and Western blot **c**. The promoter binding sites of acetylation-histone 3 and 4 were determined by ChIP; M: DNA marker; ND: No DNA control; NC: Negative control; PC: Positive control **d**. Results are representative of 3 different experiments, and the data are expressed as mean ±standard deviation with the control as “1”. Note: S3, SAHA 3 μM; M0.3, MG132 0.3 μM; S6, SAHA 6 μM; M0.6, MG132 0.6 μM; S3 + M0.3, SAHA 3 μM and MG132 0.3 μM. *p < 0.05, vs treatment groups.

After the treatment with SAHA or/and MG132, the mRNA expression levels of *p21, p27, Cdc25C, Cyclin B1, Cyclin D1, Cdc2, c-myc, Bcl-2, MMP9* and *Nanog* in U87 and U251 cells were lower than those observed in the control, while the mRNA expression levels of *p53, 14-3-3* and *Bax* were higher than those of control cells (Figure 4a, 4b, p<0.05). According to Western blot, SAHA or/and MG132 exposure up-regulated the expression of acetyl-histones 3 and 4, p53, 14-3-3 and Bax proteins, and down-regulated the expression of p21, p27, Cdc25C, Cyclin B1, Cyclin D1, Cdc2, c-myc, Bcl-2 and MMP9 in both cell lines (Figure 4c).

ChIP assay results indicated that acetyl-histone 3 and 4 bound to the *p21, p27, Cyclin D1, c-myc* and *Nanog* promoters. The binding acetyl-histone 3 and 4 to *p21, p27, Cyclin D1, c-myc* and *Nanog* promoters were increased after glioma cells were treated with SAHA (Figure 4d).

### The association of acetyl-histone 3 and 4 expression with the pathogenesis and clinicopathological parameters of glioma

**Figure 5:**
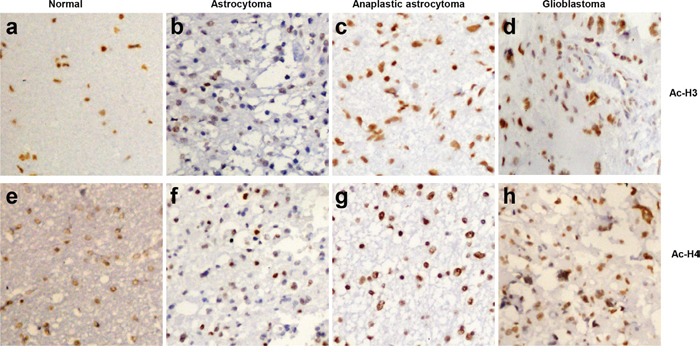
The expression of acetyl-histones 3 and 4 in gliomas Acetyl-histones 3 and 4 proteins were analyzed by immunohistochemistry (**200×**) in normal brain tissues **a, e**. astrocytoma **b, f**. anaplastic astrocytoma **c, g**. and glioblastoma **d, h**.

Acetyl-histones 3 and 4 were positively expressed in glioma tissues (Figure 5b-5d, 5f-5h) and normal brain tissues (Figure 5a, 5e). Based on the expression frequency and density, acetyl- histones 3 and 4 were found to be higher expressed in glioma tissues than normal brain tissues (Table 1, p<0.05). There was a close correlation between acetyl-histone 4 expression and several clinicopathological factors including sex, age and histological subtyping (astrocytoma, anaplastic astrocytoma and glioblastoma) in gliomas (Table 2, p<0.05).

**Table 1:** The acetyl-histones 3 and 4 expression levels in glioma tissues

Groups	acetyl-histone 3 expression							acetyl-histone 4 expression						
	n	-	+	++	+++	PR(%)	*p*	n	-	+	++	+++	PR(%)	*p*
**Normal**	33	2	4	17	10	93.9	0.048*	14	3	5	5	1	78.6	<0.001*
**Cancer**	187	9	22	57	99	95.2		40	1	7	8	24	97.5	

* p < 0.05, compared to normal brain tissues; PR, positive rate

**Table 2:** Relationship between acetyl-histones 3 and 4 expression and the clinicopathological features of glioma

Clinicopathological features	acetyl-histone 3 expression							acetyl-histone 4 expression						
	n	-	+	++	+++	PR(%)	p	n	-	+	++	+++	PR(%)	p
**Sex**														
Male	111	9	14	31	57	91.9	0.229	111	13	19	33	46	88.3	0.014*
Female	75	0	8	25	42	100		76	1	10	23	42	98.7	
**Age (years)**														
<44	83	4	8	25	46	95.2	0.482	85	7	18	30	30	91.8	0.004*
≥44	102	5	14	31	52	95.1		101	7	11	25	58	93.1	
**Pathological grading**														
I-II	81	3	7	31	40	96.3	0.260	84	7	14	34	29	91.7	0.112
III-IV	51	5	10	12	24	90.2		47	6	6	8	27	87.2	
**Pathological classification**														
astrocytoma	102	8	12	33	49	92.2	0.384	103	11	20	33	39	89.3	0.004*
anaplastic astrocytoma	32	1	4	7	20	96.9		31	2	3	7	19	93.5	
glioblastoma	26	0	5	8	13	100		25	0	3	7	15	100	

* p< 0.05; PR, positive rate

### SAHA and/or MG132 inhibits glioma growth *in vivo*

**Figure 6:**
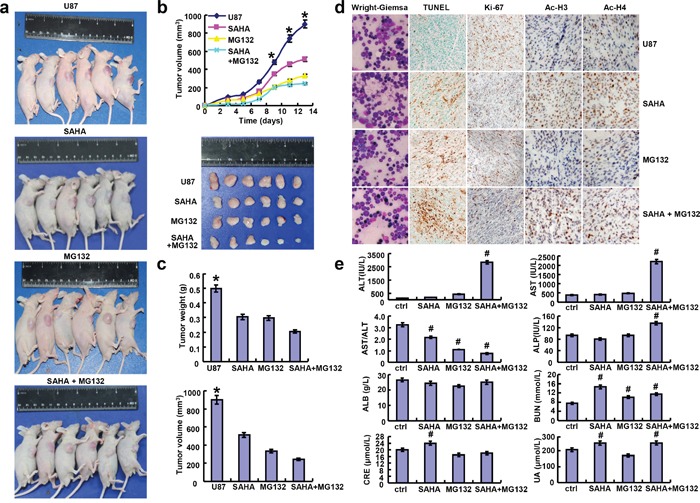
SAHA or/and MG132 suppressed the growth of glioma cells *in vivo* After the exposure to SAHA and/ or MG132, the growth of U87 cells was revealed by measurement of tumor size **a**. and growth curve **b**. volumeand weight **c**. Wright-Giemsa staining was used to observe the morphological appearance of bone marrow. Stronger acetyl-histones 3 and 4 staining was observed in SAHA group than the control **d**. Either weaker ki-67 or stronger TUNEL staining was seen in SAHA or/and MG132 group than the control (d). The serum levels of ALT, AST, ALP, ALB, BUN, CRE and UA were measured by automatic biochemical analyzer **e**. Note: SAHA, 20 mg/kg; MG132, 2 mg/kg; SAHA+MG132, SAHA 20 mg/kg and MG132 2 mg/kg. *p< 0.05, vs treatment groups; **^#^**p < 0.05, vs control group.

The tumor volumes of U87 xenografts became smaller than the control after the treatment with SAHA or/and MG132 by calculation and weighting (Figure 6a-6c, p<0.05). The suppressing effect was positively correlated with a low proliferation and a high apoptosis in comparison to the control by ki-67 immunostaining and TUNEL assay (Figure 6d). After treatment with SAHA, glioma cells showed a higher expression of acetyl-histones 3 and 4 than the control (Figure 6d).

According to bone marrow staining, we found that SAHA or/and MG132 didn't suppress the hematopoiesis (Figure 6d). However, SAHA or MG132 exposure increased the serum level of blood urea nitrogen (BUN), and the combination of both reagents had a worse effect on alanine aminatransferase (ALT), aspartate aminotransferase (AST) and alkaline phosphatase (ALP), but the converse was true for AST/ALT. There appeared a high level of creatinine (CRE) and uric acid (UA) in tumor-bearing nude mice, exposed to SAHA alone (Figure 6e, p<0.05).

## DISCUSSION

The anti-tumor effects of SAHA have been reported in chronic myelogenous leukemia [7], lung [8], pancreatic [9], liver [10], cervical [11], head and neck [12], breast [13] and ovarian cancers [14]. In a clinical phase I study of advanced leukemias and myelodysplastic syndrome, a significant antileukemia activity was observed at or below maximum tolerated dose, and SAHA effectively inhibited HDAC activity in peripheral blood and bone marrow blasts [15]. Reportedly, co-administration of ABT-737 and SAHA can induce apoptosis in PTEN-intact malignant human glioma cells [16]. Continuous intracranial administration of SAHA inhibits tumor growth in an orthotopic glioma model [17]. Glutamate transporter xCT promotes glioma progression and SAHA specifically inhibits the xCT transporter expression to normalize the glioma microenvironment [18]. Here, we found that SAHA inhibited cell viability, energy metabolism of both cells lines, induced apoptosis, G_2_ arrest and inhibited migration, invasion and the ability to form lamellipodia in U87 and U251 glioma cells when used alone. The combination of MG132 and SAHA exhibited an additive effect, suggesting that this dual effect may more effective in the treatment of glioma than SAHA alone.

In the present study, we for the first time found that SAHA or/and MG132 might remarkably inhibit glioma growth in tumor-bearing mice with no marrow toxicity, but hepatic and renal toxicity occurred. Furthermore, their inhibitory effects were closely linked to lower proliferation and higher apoptosis. Reportedly, the combination of SAHA and XL184 synergistically induced cell apoptosis and inhibited tumor growth of prostate and lung cancer cells [19]. The similar results were observed in gastric [20] and hepatocellular [21] cancer cells, treated by oxaliplatin and SAHA. Therefore, they might be employed to treat the glioma if both hepatic and renal injuries are avoided and ameliorated in clinical practice.

Both p21 and p27 can interact with cyclin-CDK complex to arrest G_1_ phage of cell cycle. SAHA or/and MG132 treatment resulted in significant decreases in mRNA and protein expression of p21 and p27 in both glioma cells, suggesting that SAHA down-regulated p21 and p27 for G_2_ arrest [22]. *Cdc25C* gene contains tumor suppressor *p53* binding sites and is demonstrated to contribute to the p53-dependent cell cycle arrest [23]. Cyclin B1 is involved in the early events of mitosis by interacting with Cdk1, and Cdc2 is an M-phase promoting factor that induces entry into mitosis [24–26]. Our results showed that the mRNA and protein expression of p53 were significantly increased by SAHA or/and MG132 treatment, but Cdc2, Cyclin B1 and Cdc25C decreased, indicating their roles in G_2_ arrest. Cell apoptosis was induced by the translocation of Bax to mitochondria through phosphorylation of 14-3-3 [27]. SAHA or/and MG132 induced apoptosis in the glioma cells was attributable to the down-regulation of Bcl-2 protein levels and the up-regulation of 14-3-3, Bax, Caspase 9, Capase 3 expression via extrinsic and intrinsic pathways [28, 29]. Our results also showed that SAHA or/and MG132 decreased expression of MMP9 and Nanog in both glioma cells. MMP9 overexpression enhances cancer metastasis via breakdown of the extracellular matrix [30]. Nanog is a stem cell transcription factor that is essential for malignant transformation and progression [31]. It has been reported that down regulation of Nanog by histone deacetylase inhibitor could lead to cell cycle arrest, differentiation and apoptosis in human embryonic carcinoma NCCIT cells [32].

Here, we performed the ChIP assay with the antibodies against acetyl-histone 3 and 4 targeting the promoters of *p21, p27, Cyclin D1, c-myc* and *Nanog*. It was found that SAHA promoted acetylation of histones 3 and 4 in glioma cells, which were recruited to the promoter of *p21, p27, Cyclin D1, c-myc* and *Nanog* for their down-regulated transcription. To investigate the clinicopathological significance of both of these acetyl-histone proteins, we carried out immunohistochemistry analysis. There showed a significantly higher expression of both acetyl-histone 3 and 4 in gliomas than in normal brain tissues. These findings suggest that the overexpression of both the proteins may be a reactive response in glioma. Additionally, acetyl-histone 4 expression was positively correlated with histological aggressiveness of glioma in line with the data from renal cell carcinomas [33]. In our previous study, we found that acetyl-histone 4 expression was highly expressed in more poorly- differentiated ovarian cancer in agreement with this finding [34]. Therefore, the expression of acetyl-histones 3 and 4 would be potential marker to monitor the efficacy of SAHA as described in peripheral blood mononuclear cells [15].

In line with our results, it was found that the combination of SAHA with cisplatin or decitabine could enhance the anti-tumor effect in oral squamous cell carcinoma [35] and a clinical study [36]. The combination of MG132 with cisplatin or arsenic trioxide could increase sensitivity to drug therapy in ovarian cancer [37] or Raji cells [38]. But the serum toxicity of combined drugs in nude mice was not reported. In agreement with the result from O'Connor et al. [39], our study showed that the nude mice serum analysis showed that the level of CRE, UA and BUN were higher exposed to SAHA alone. Therefore, they can be employed to kill or inhibit cancer cell growth at an additive manner, but its safety and effectiveness should be monitored in clinical practice.

In conclusion, SAHA and MG132 *in vitro* and vivo have an additive effect in glioma cell lines by inducing apoptosis, G_2_ arrest, and suppressing proliferation, energy metabolism, migration, invasion, lamellipodia formation and tumor growth. SAHA may increase the expression of acetyl-histones 3 and 4 and thereby down-regulate the mRNA expression of downstream genes, including *p21, p27, Cyclin D1, c-myc* and *Nanog*. Overexpression of these acetyl-histones may be positively linked to the tumorigenesis and differentiation of glioma. Therefore, SAHA or/and MG132 could potentially be employed as a chemotherapeutic agent in clinical practice, but we must prevent the hepatic and renal toxicity in glioma treatment.

## MATERIALS AND METHODS

### Cell culture

Glioma cells U87 and U251 were obtained from ATCC (Manassas, VA, USA) and grown in a humidified atmosphere of 5% CO_2_ at 37 °C in MEM (U87) or DMEM (U251) with 10% fetal bovine serum (FBS), 100 U/ml penicillin and 100 μg/ml streptomycin added. All cells were collected by centrifugation, washed in phosphate buffered saline (PBS) and subjected to RNA and protein extraction. We treated glioma cells to SAHA (Sigma, USA) and MG132 (Sigma, USA) for the following experiments.

### Proliferation assay

Cells were planted at 2.0×10^3^ cells per well in 96-well plates and maintained in media containing 10% FBS. At the time points, 20 μl of 5 mg/ml MTT was added to each well, and then incubated for 4 h at 37 °C. After that, the media was discarded, and 150 μl of DMSO was added to each well to dissolve the precipitates. The absorbance was measured at 490 nm using a microplate reader (M200pro, Switzerland).

### Cellular energy metabolism

Mitochondrial respiration and glycolysis were measured by Seahorse XF Extracellular Flux Analyzer. Respiration and glycolysis are respectively measured as the rate of oxygen consumption (OCR) and extracellular acidification (ECAR). According to the standard protocol, we injected the regents in the following order: 10 mM glucose, 1.5 μM oligomycin (which blocks ATP synthase to assess respiration required for ATP turnover), 50 mM 2-DG for glycolysis assay and 1.5 μM oligomycin, 2 μM FCCP (a proton ionophore which induces chemical uncoupling and maximal respiration), 0.5 μM rotenone and antimycin A (which completely inhibits electron transport to measure non-mitochondrial respiration) for mitochondrial respiration. OCR and ECAR values were determined from 3 wells per sample and experiments were replicated.

### Cell cycle analysis

After the treatment of reagents, each group cells were washed twice with PBS, and then detached by trypsinization. Cells were collected and added with 1 ml precool 70% ethanol for at least 2 h. The cells were washed twice with PBS again and incubated for 1 h at 37°C with 1 ml RNase (0.25 mg/ml). The cells were resuspended in propidium iodide (PI) at a concentration of 50 μg/ml and cycle analysis was performed by flow cytometry.

### Apoptosis assay

The apoptosis rate was determined by using PI and FITC-labeled annexin V (*KeyGEN* Biotech, Nanjing, *China*) following the manufacturer's instruction. In brief, cells were washed twice with ice-cold PBS, resuspended in 200 μl 1×binding Buffer at a concentration of 1×10^6^ cells/ml, incubated with 10 μl FITC-Annexin V for 15 min in the dark and then resuspended in 300 μl 1×bingding buffer with 5 μl PI added. Flow cytometry was performed within 1 h of incubation and apoptosis was analyzed by FlowJo software.

### Immunofluorescence

2.0×10^3^ cells were planted in 6-well plates with glass coverslips, fixed with 4% paraformldehyde for 15 min and permeabilized with 0.5% Triton X-100 in PBS for 15 min at room temperature. After washed with PBS, the cells were incubated overnight at 4°C with 5 μg/ml Phalloidin (Invitrogen, Carlsbad, CA, USA). Nuclei were stained with 1 mg/ml DAPI (Sigma, USA) for 2-3 min at room temperature. The coverslips were then mounted with anti-fade mounting medium and observed under a confocal laser microscope.

### Wound healing assay

Cell migration was assessed using a wound healing assay as described previously [34]. Briefly, cells of 6×10^5^ were seeded into 6-well plate and incubated overnight. A scratch wound was made with a tip. Cells were washed three times with PBS, and cultured in FBS-free media, photographed after 24 h. The scratch area was measured using Image pro-Plus 6.0.

### Cell invasion assay

Cell invasion assay was conducted using a matrigel-coated transwell inserts. 2.5×10^5^ cells were resuspended in 200 μl serum-free MEM (U87) or DMEM (U251) and planted to each insert. 600 μl of media was added to the lower chambers contained 10% FBS. After incubation at 37 °C and 5% CO_2_ for 48 h, the cells on the upper surface of the inserts were wiped away with a cotton swab. The cells on the lower surface of the membrane were washed twice with PBS, fixed with 4% paraformaldehyde for 15 min, washed with PBS again, and stained with crystal violet dye (0.1%) for the measurement.

### Real-time reverse transcriptase–polymerase chain reaction (RT-PCR)

Total RNA was isolated from glioma cells after 48 h of treatment using Trizol (Takara, Japan) and quantified by a Nanodrop spectrophotometer (Wilmington, USA). Reverse transcription was performed from 2 μg of total RNA using AMV reverse transcriptase and random primers (Takara, Japan). PCR primers were designed according to the sequences in GenBank and are listed in Table 3. Amplification of cDNA was performed using SYBR Premix Ex Taq II kit (Takara, Japan) using *GAPDH* as an internal control.

**Table 3:** Primer sequences selected for real-time RT-PCR

Name	Primer sequence	Distribution	AT(°C)	Product size (bp)	Extension time (s)
*p53*	F: 5' - ACCTATGGAAACTACTTCCTGA - 3' R: 5' - TGGCATTCTGGGAGCTTCA - 3'	NM_001276696.1264-403	60	139	39
*p21*	F: 5' - ACTGTCTTGTACCCTTGTGCC - 3' R: 5' - AAATCTGTCATGCTGGTCTGC - 3'	XM_003950827572-679	60	108	39
*pP27*	F: 5' - GGCTCCGGCTAACTCTGA - 3' R: 5' - TTCTTCTGTTCTGTTGGCTCTT - 3'	XM_5223471081-1237	60	157	39
*Cdc25C*	F: 5' - ACTGGTCACCTGGATTCTT - 3' R: 5' - AAACCATTCGGAGTGCTAC - 3'	XM_011543763.119-131	60	113	39
*Cyclin B1*	F: 5' - GTTATGCAGCACCTG - 3' R: 5' - CTTGGCTAAATCTTGAACT - 3'	NM_0010885901388-1537	60	150	39
*Cyclin D1*	F: 5' - TGCCACAGATGTGAAGTTCATT - 3' R: 5' - CAGTCCGGGTCACACTTGAT - 3'	NG_000002776-937	60	162	39
*Cdc2*	F: 5' - GGGCACTCCCAATAA - 3' R: 5' - GATGCTAGGCTTCCTG - 3'	XM_572099631-723	60	93	39
*c-myc*	F: 5' - AGCGACTCTGAGGAGGAACA - 3' R: 5' - TCCAGCAGAAGGTGATCCA - 3'	XM_006761318-1425	60	108	39
*14-3-3*	F: 5' - CAAAGACAGCACCCTCA - 3' R: 5' - TTCTGCCGCATCACAT - 3'	XM_010379682.1845-935	60	91	39
*Bax*	F: 5' - GATTGCCGCCGTGGAC - 3' R: 5' - GCCCCAGTTGAAGTTGC - 3'	DQ926869306-393	60	88	39
*Bcl-2*	F: 5' - GCCTTCTTTGAGTTCGGTGGG - 3' R: 5' - TGTGCAGGTGCCGGTTCAG - 3'	DQ926871938-1052	60	115	39
*MMP9*	F: 5' - TGTACCGCTATGGTTACACT - 3' R: 5' - CCTCAAAGGTTTGGAAT - 3'	KJ897197.1211-399	60	189	39
*Nanog*	F: 5' -CAAAGGCAAACAACCCACTT- 3' R: 5' -TCTGCTGGAGGCTGAGGTAT- 3'	NM_024865.2432-589	60	158	39
*GAPDH*	F: 5' - CAATGACCCCTTCATTGACC - 3' R: 5' - TGGAAGATGGTGATGGGATT - 3'	NM_ 002046.3201-335	60	135	39

Abbreviation: AT, annealing temperature

### Western blot analysis

Cells were washed with cold PBS twice and sonicated in RIPA lysis buffer. Protein assays were performed by Kaumas brilliant blue method, resolved in 10% SDS-PAGE and electrotransferred to a PVDF membrane. The membrane was blocked with 5 % skim milk in Tris-buffered saline with Tween 20 for 1 h at room temperature and incubated with the primary antibodies (Table 4) on the shaker at 4 °C overnight. The membranes were rinsed with TBST, and incubated with anti-rabbit, anti-mouse or anti-goat IgG antibody conjugated to horseradish peroxidase (HRP, Dako, USA). Bands were visualized with LAS4010 (GE healthcare Life Science, USA) by ECL-Plus detection reagents (Santa Cruz, USA). Densitometric quantification of protein bands was performed with GAPDH as a control using Image J (NIH, USA).

**Table 4:** Antibodies used for western blot

Name	Source	Dilution	Company
Ac-histone 3 (Lys 9/14)	Goat	1:500	Santa Cruz Biotechnology
Ac-histone 4 (Lys 8)	Rabbit	1:500	Santa Cruz Biotechnology
p53	Rabbit	1:1000	Wanleibio
p21 (F-5)	Mouse	1:500	Santa Cruz Biotechnology
p27 (C-19)	Rabbit	1:500	Santa Cruz Biotechnology
Cdc25C	Rabbit	1:1000	Wanleibio
Cyclin B1	Rabbit	1:1000	Wanleibio
Cyclin D1 (H-295)	Rabbit	1:500	Santa Cruz Biotechnology
Cdc2 (C-9)	Mouse	1:500	Santa Cruz Biotechnology
c-myc (9E10)	Mouse	1:500	Santa Cruz Biotechnology
14-3-3 (H-8)	Mouse	1:500	Santa Cruz Biotechnology
Bax (B-9)	Mouse	1:500	Santa Cruz Biotechnology
Bcl-2 (C21)	Rabbit	1:300	Santa Cruz Biotechnology
MMP9	Rabbit	1:1000	Wanleibio
ki-67	Rabbit	1:80	Abcam
GAPDH	Rabbit	1:2000	Wanleibio

### Chromatin immunoprecipitation

ChIP assays were performed with ChIP assay kit (MILLIPORE Magna ChIP^TM^ G). U87 and U251 cells were fixed with 1% formaldehyde for 10 min and quenched with 0.25 mM glycine. Immunoprecipitation was performed with the following antibody against acetyl-histone H3 (Lys 9/14, Santa Cruz, USA), acetyl-histone H4 (Lys 8, Santa Cruz, USA), RNA Polymerase II (05-623B, MILLIPORE) or normal mouse IgG (12-371B, MILLIPORE). DNA was purified using spin columns. PCR reaction was performed as follows: initial denaturation at 95 °C for 5 min followed by 30 cycles of denaturation at 95 °C for 30 s, then annealing for 30 s and extension at 72 °C for 30 s. PCR primers are listed in Table 5.

**Table 5:** Primer sequences for ChIP

Name	Primer sequence	AT (°C)	Product size (bp)	Extension time (s)
*p21-676*	F:5'-CCCGGAAGCATGTGACAATC-3' R:5'-CAGCACTGTTAGAATGAGCC-3'	56	354	30
*p21-981*	F:5'-GGAGGCAAAAGTCCTGTGTTC-3' R:5'-GGAAGGAGGGAATTGGAGAG-3'	56	306	30
*p21-2036*	F:5'-GGAGTCAGATTCTGTGTGTG-3' R:5'-CCTCTGCTTTCAGGCATTTC-3'	56	368	30
*Cyclin D1-704*	F:5'-TGAAAATGAAAGAAGATGCAGTCG-3' R:5'-CTGTAGTCCGGTTTTCATAGAAATGC-3'	57	328	30
*Cyclin D1-738*	F:5'-GTCCTACTTCAAATGTGTGCAGAAGG-3' R:5'-CTCCCACGAAACGCTACTTCTAGC-3'	57	290	30
*Cyclin D1-744*	F:5'-CCCAGTTACTGTCGTTATCTCTCATC-3' R:5'-ATCCCTTTTGTAGCATCCCAAGAG-3'	57	294	30
*c-myc-2k*	F:5'-TCACGTTTGCCATTACCGGTTC -3' R:5'-TTTCAGGTTGGCTGCA G A AGGT-3'	58	171	30
*c-myc-77*	F:5'-CAGGGCTTCTCAGAGGCTTGG-3' R:5'-CTGCTCGCCCGGCTCTTCC ACC-3'	58	162	30
*c-myc-1572*	F:5'-CAGATCAGCAACAACCG AAA-3' R:5'-GGCCTTTTCATTGTTTTCCA-3'	58	167	30
*p27*	F: 5' -CTGTCACATTCTGGAGCGTA- 3' R: 5' -AGTGGATCTTCAACTGCCTC- 3'	60	230	30
*Nanog*	F: 5' -GTTCTGTTGCTCGGTTTTCT- 3' R: 5' -TCCCGTCTACCAGTCTCACC- 3'	60	95	30
*Poly II*	F: 5' -TACTAGCGGTTTTACGGGCG- 3' R: 5' -TCGAACAGGAGGAGCAGAGAGCGA- 3'	59	116	30

Abbreviation: AT, annealing temperature

### Selection of patient samples

A total of 220 glioma cases were collected from surgical resection in The First Affiliated Hospital of Jinzhou Medical University (n=100) from 2002 to 2014, and Shengjing Hospital of China Medical University (n=120) from 2007 to 2014. The average age of the patients at surgery was 43.4 years (2-80 years). The majority of samples were routinely prepared for storage in pathological blocks. None of the patients had undergone chemotherapy, radiotherapy or adjuvant treatment prior to surgery. Informed written consent was obtained from all participants and the study was approved by Ethics Committees of both Universities.

### Pathology and tissue microarray (TMA)

All tissues were fixed in 10% neutral formalin, embedded in paraffin and sliced into 4 μm- thick sections. The sections were stained with hematoxylin-and-eosin (HE) for histological analysis. The clinicopathological staging was evaluated for each glioma sample according to TNM staging system. Tumor histology was determined according to World Health Organization classification system.

Representative areas of solid tumors were identified in HE-stained slices of selected tumor samples and 2mm-in-diameter tissue cores were punched out from each donor block and transferred to a recipient block using a Tissue Microarrayer (AZUMAYA KIN-1, Tokyo, Japan). Consecutive 4μm sections were incised from the recipient block and transferred to poly-lysine-coated glass slides.

### Xenograft models

Balb/c nude mice of 6-8 weeks were kept in a specific pathogen-free (SPF) facility with a 12 h light/dark cycle. The experiments were carried out in accordance to Animal Experiment Ethical Statement. All experimental protocols were approved by The First Affiliated Hospital of Jinzhou Medical University Ethics Committee.

U87 cells were detached by trypsinization, washed and re-suspended in serum-free medium. Subcutaneous xenografts were established by injection of 1× 10^6^ cells per mouse to axilla (n=6 mice/group). Until tumor diameter reached 8 mm, we began to intraperitoneally inject 20 mg/kg SAHA, 2 mg/kg MG132 and 20 mg/kg SAHA + 2 mg/kg MG132 into mice from 8th, 10th, and 12th day of cell injection. Tumor growth was then monitored for 13 days and calculated as follows: length×width×width×0.5. At the end of the experiment, mice from each group was anesthetized, photographed, and sacrificed for further analysis.

In brief, the peripheral blood of nude mice was collected from abdominal vein after being killed by cervical dislocation, kept into a venous blood sample collection vessel, and centrifuged at 4000 rpm per minutes for 5 min. After that, the supernatant was analyzed for alanine aminatransferase (ALT), aspartate aminotransferase (AST), alkaline phosphatase (ALP), albumin (ALB), blood urea nitrogen (BUN), creatinine (CRE) and uric acid (UA) by automatic biochemical analyzer (Hitachi 7600, Japan). The bone marrow was taken out from femurs of nude mice for Giemsa-Wright staining.

### Immunohistochemistry (IHC)

IHC was carried out on consecutive 4 μm-thick sections using intermittent microwave irradiation two-step method [40]. The samples were deparaffinized with xylene three times, rehydrated with alcohol, and subjected to antigen retrieval by heating in target retrieval solution (TRS, Dako, Japan) for 20 min in a microwave oven. The sections were quenched with 3% hydrogen peroxide for 5 min to block endogenous peroxidase activity. Non-specific binding was prevented by adding 5% bovine serum albumin for 5 min. The sections were incubated with rabbit anti-acetyl-histone-3 (1:200, Lys 9/14, Santa Cruz, USA), rabbit anti-acetyl-histone H4 (1:200, Lys 8, Santa Cruz, USA) or rabbit anti-ki-67 (1:80, Abcam, UK) for 2 h, then incubated with anti-rabbit antibody conjugated to HRP (Dako) for 1 h. After each treatment, all sections were washed three times with TBST and the binding sites were visualized with diaminobenzidine (DAB). After counterstained with hematoxylin, the sections were dehydrated, cleared and mounted. Two independent observers (YXF and ZZJ) randomly selected five representative fields from each section. Any discrepancies were checked by both observers until a consensus was reached. Positive expression was graded as follows: “–” = negative, “+” = 1–50%, “++” = 50–74%, “+++” = ≥ 75%.

### Terminal deoxynucleotide transferase mediated dUTP nick labeling (TUNEL)

TUNEL was performed using Apoptosis Detection Kit (Millipore, USA). According the manufacturer's instruction, paraffin-embedded sections were incubated with proteinase K at 37 °C for 30 min. Endogenous peroxidase activity was blocked by incubation with 3 % hydrogen peroxide in methanol. The sections were washed three times with PBS then subjected to TUNEL staining. The conjugated horseradish peroxidase was visualized with DAB, followed by counterstaining with methyl green.

### Statistical analysis

Statistical evaluation was performed using Spearman's correlation coefficient to analyze ranked data, and Mann-Whitney U to differentiate the means of different groups. The correlations were performed using linear regression analysis. A p-value < 0.05 was considered as statistically significant. SPSS 10.0 software was employed to analyze all data.
